# Improving the quality of a collective signal in a consumer EEG headset

**DOI:** 10.1371/journal.pone.0197597

**Published:** 2018-05-24

**Authors:** Alejandro Morán, Miguel C. Soriano

**Affiliations:** Instituto de Física Interdisciplinar y Sistemas Complejos, IFISC (CSIC-UIB), Campus Universitat Illes Balears, Palma de Mallorca, Spain; Ghent University, BELGIUM

## Abstract

This work focuses on the experimental data analysis of electroencephalography (EEG) data, in which multiple sensors are recording oscillatory voltage time series. The EEG data analyzed in this manuscript has been acquired using a low-cost commercial headset, the Emotiv EPOC+. Our goal is to compare different techniques for the optimal estimation of collective rhythms from EEG data. To this end, a traditional method such as the principal component analysis (PCA) is compared to more recent approaches to extract a collective rhythm from phase-synchronized data. Here, we extend the work by Schwabedal and Kantz (PRL 116, 104101 (2016)) evaluating the performance of the Kosambi-Hilbert torsion (KHT) method to extract a collective rhythm from multivariate oscillatory time series and compare it to results obtained from PCA. The KHT method takes advantage of the singular value decomposition algorithm and accounts for possible phase lags among different time series and allows to focus the analysis on a specific spectral band, optimally amplifying the signal-to-noise ratio of a common rhythm. We evaluate the performance of these methods for two particular sets of data: EEG data recorded with closed eyes and EEG data recorded while observing a screen flickering at 15 Hz. We found an improvement in the signal-to-noise ratio of the collective signal for the KHT over the PCA, particularly when random temporal shifts are added to the channels.

## Introduction

Non-invasive techniques such as electroencephalography (EEG), functional magnetic resonance imaging (fMRI) or magnetoencephalography (MEG) are widely used to study the brain activity [1–3]. Since EEG devices are more portable than MEG and have better time resolution than fMRI, they are being used in many different clinical and research environments [4, 5]. Accordingly, there is a wide range of prices for EEG devices, from brain—computer interface systems designed for a specific task to medical-grade devices with hundreds of high quality electrodes. These measurement devices are all based on the same principle, neurons communicate through chemical neurotransmitters and electrical impulses, giving rise to electromagnetic waves. Electrodes are then used in EEG to measure oscillatory signals related to action potential across different regions of the brain. In EEG devices, it is generally believed that most of the measured signal is provided by pyramidal neurons of the cortex [6, 7].

Consumer EEG headsets are typically used for gaming and simple brain-computer interface (BCI) tasks. However, these consumer devices are being used more and more in research [8–11]. Low-cost EEG devices suffer from several impairments compared to medical-grade ones, including a lower signal quality and an imprecise timing. Recent work by Matthieu Duvinage et al. suggests that the signal to noise ratio (SNR) is a weak point of these apparatus in comparison to that present in medical-grade systems [12]. Other aspects such as maintenance costs and patient comfort are also relevant in the comparison between devices. Here, we focus on the SNR improvement of the EEG signal from a consumer EEG headset but we do not compare directly to medical-grade devices. In this context, improving the SNR could make some consumer headsets a reasonable alternative to the medical ones for non-critical tasks.

The raw data measured in EEG is oscillatory, and it is common to examine the data for different frequency bands [13]. A commonly studied frequency band is the alpha band, which corresponds to neural oscillations in the frequency range of 8–13 Hz. In adults, the activity in this band is present being awake with the eyes open, and is strongly amplified when our eyes are closed [14]. Alpha brain waves are also present in some kinds of sleep, reversible coma or migraine [15]. Other frequency bands of interest include e.g. delta (0.2–3 Hz), theta (4–7 Hz), beta (13–30 Hz) and gamma (30–70 Hz) brain waves.

The brain activity may exhibit characteristic frequencies for certain tasks, e.g. memory retrieval or sustained attention [16]. In this context, phase synchronization has been shown to be a good indicator to characterize normal brain function [17, 18]. In particular, memory-related operations result in a high degree of phase synchronization in the theta and gamma bands [19, 20]. This mechanism is thought to facilitate communication between brain regions. Phase synchronization has also been used as a tool to characterize brain pathologies [18]. Abnormal phase synchronization properties have been observed in the case of schizophrenia disorders in the gamma band [21] or in epilepsy [22]. In the case of phase synchronization, a collective rhythm provides valuable information about the activity of the brain in specific frequency bands. A collective rhythm can be obtained when all the signals measured in different EEG channels are compressed in a single one, obtained as a combination of the individual recordings. Since EEG recordings consist of multiple and simultaneous measurements of brain oscillations from different locations, they can be interpreted as a set of measurements from noisy nonlinear coupled oscillators [23]. Thus, techniques originating from the study of nonlinear dynamical systems prove valuable in the extraction of a collective brain rhythm [24–27].

Our work focuses on the analysis and comparison of different techniques for the estimation of collective rhythms from EEG data measured using a consumer EEG device. More specifically, we choose the Emotiv EPOC+ for our measurements as it is a user-friendly, comfortable and non-invasive EEG headset with 14 channels. In what follows, we describe the different phase extraction methods and compare their benefits. We show that the Kosambi-Hilbert Torsion (KHT) method [27] is extremely effective at estimating a global signal from EEG data. We evaluate the performance for two sets of measurements: EEG data recorded in a resting state with eyes closed and EEG data recorded while observing a screen flickering at 15 Hz. For these sets of data, we expect, respectively, the observation of strong brain activity between 8 and 12 Hz with a maximum near 10 Hz (alpha rhythm), and phase locking in a narrow range of frequencies with a maximum around 15 Hz (screen flickering rate).

## Materials and methods

Since we intend to improve the SNR of collective oscillations in commercial devices, we first describe the specifics of the EEG device and the algorithms employed to infer such collective oscillations. In particular, we utilized an Emotiv EPOC^®^ headset as the EEG recording device. For the extraction of a collective phase, we describe the methods of principal component analysis (PCA) [28], phaser [29], and KHT algorithms [27]. Finally, we define the notions of signal-to-noise ratio and instantaneous phase as they will be used along the manuscript.

### Emotiv

**Fig 1:**
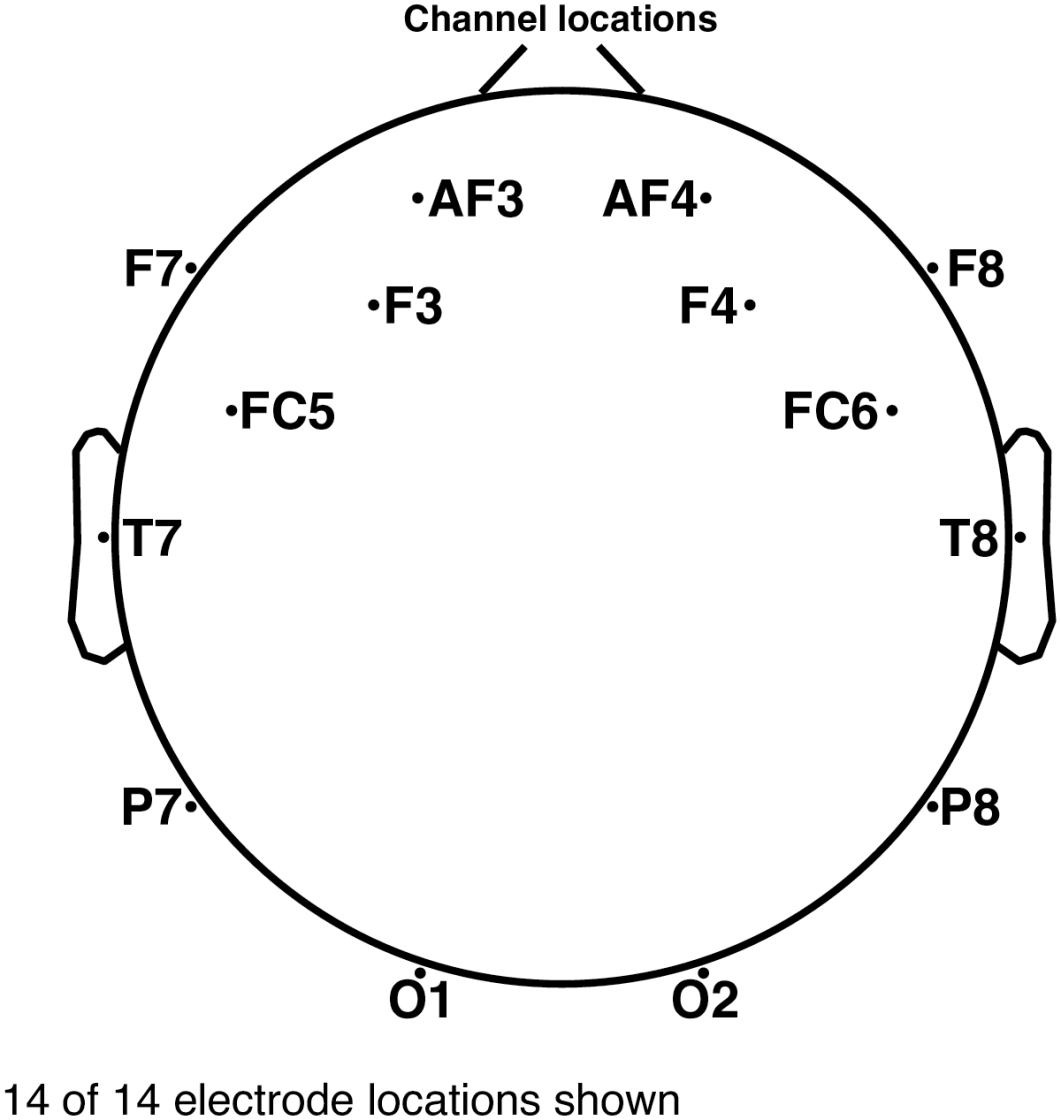
Channel locations and labels for the 14 electrodes on the Emotiv device. There are two additional reference sensors, which are 30 degrees above the ears.

A 14 channel wireless Emotiv EPOC^®^ headset has been utilized to generate the data we analyze in the present work. In this device saline based wet sensors are used to register the signal of each channel. The raw data is collected at 128 samples per second simultaneously for each channel and sent to the computer in real time via wireless transmission. Each electrode has a resolution of 0.51 *μ*V and a bandwidth of 43 Hz. In Fig 1, we provide the information about the location of the electrodes.

### Data collection

All procedures performed in studies involving human participants were in accordance with the ethical standards and with the 1964 Helsinki Declaration and its later amendments or comparable ethical standards. All participants gave informed written consent, following the ethics protocol approved by the Ethics Committee of the University of the Balearic Islands.

We have recorded our own data using the Emotiv device described above. Before placing the headset on the scalp, the electrodes are slightly wetted with a saline solution that improves skin contact (higher conductivity). Here, we have performed two types of measurements: brain activity in a resting state with closed eyes and sitting on a comfortable chair while observing a screen flickering at 15 Hz.

In the first task, the subject closes his/her eyes and brain activity is measured during 30 seconds. A similar process is repeated for the flickering task, in which the subject looks at a flashing screen with alternating colors (black and white) at a 15 Hz frequency. These tasks are repeated for five subjects to account for inter-subject variations.

In order to compare the results provided by the different methods that will be introduced, it is important to choose tasks or experiments that really test their performance in a common framework. In our case, the experiments have been chosen to test the methods on fundamentally different signals. On the one hand, the brain activity with the eyes closed presents a delocalized globally distributed oscillation around the alpha frequency band. The signal measured in this frequency range is significantly higher with the eyes closed than with the eyes opened. On the other hand, the brain activity induced by watching a flickering screen (alternating black and white colors) produces an EEG rhythm at 15 Hz within a narrow frequency range, i.e. the flicker produces a rather localized oscillation. Thus, we tested the methods on 2 different scenarios: signals with a relatively high SNR and a broad spectrum and signals with a relatively low SNR and a narrow spectrum.

We recorded several realizations for each experiment in order to obtain reliable results. In total, for each subject we recorded 6 independent realizations for the eyes closed experiment and 6 realizations for the flicker at 15 Hz in order to achieve similar relative errors for both experiments. Each realization lasts for 30 seconds.

**Fig 2:**
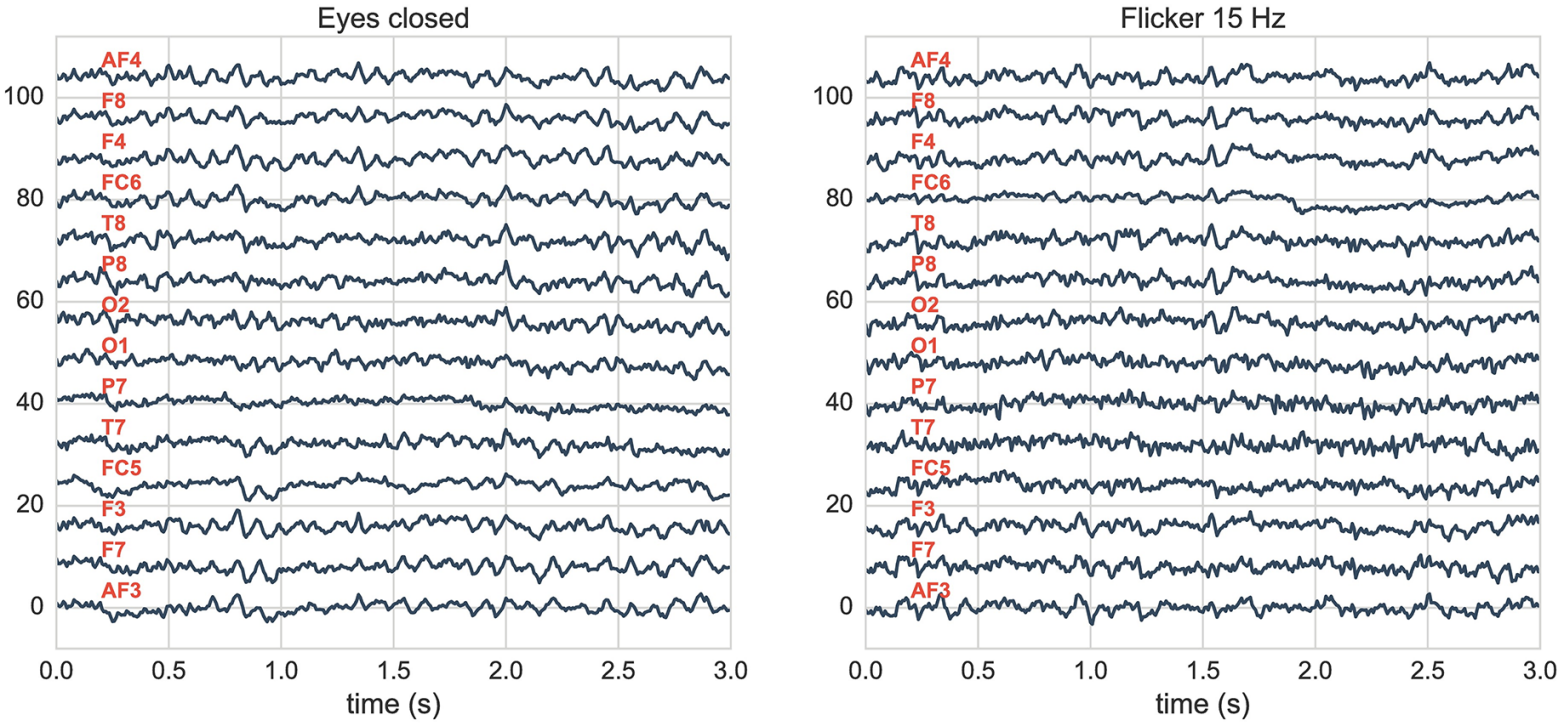
Segment of 3 seconds of normalized EEG time series for two different experiments measured using the Emotiv device. (Left) Resting state with eyes closed. (Right) Watching a screen displaying a 15 Hz flicker. (Red) Channel locations are shown in Fig 1.

After collecting the data we manually extract 10 seconds of artifact-free recordings for each subject. An example of the recorded data is shown in Fig 2, in which we can see with a naked eye that several EEG channels are highly correlated along the time series.

**Fig 3:**
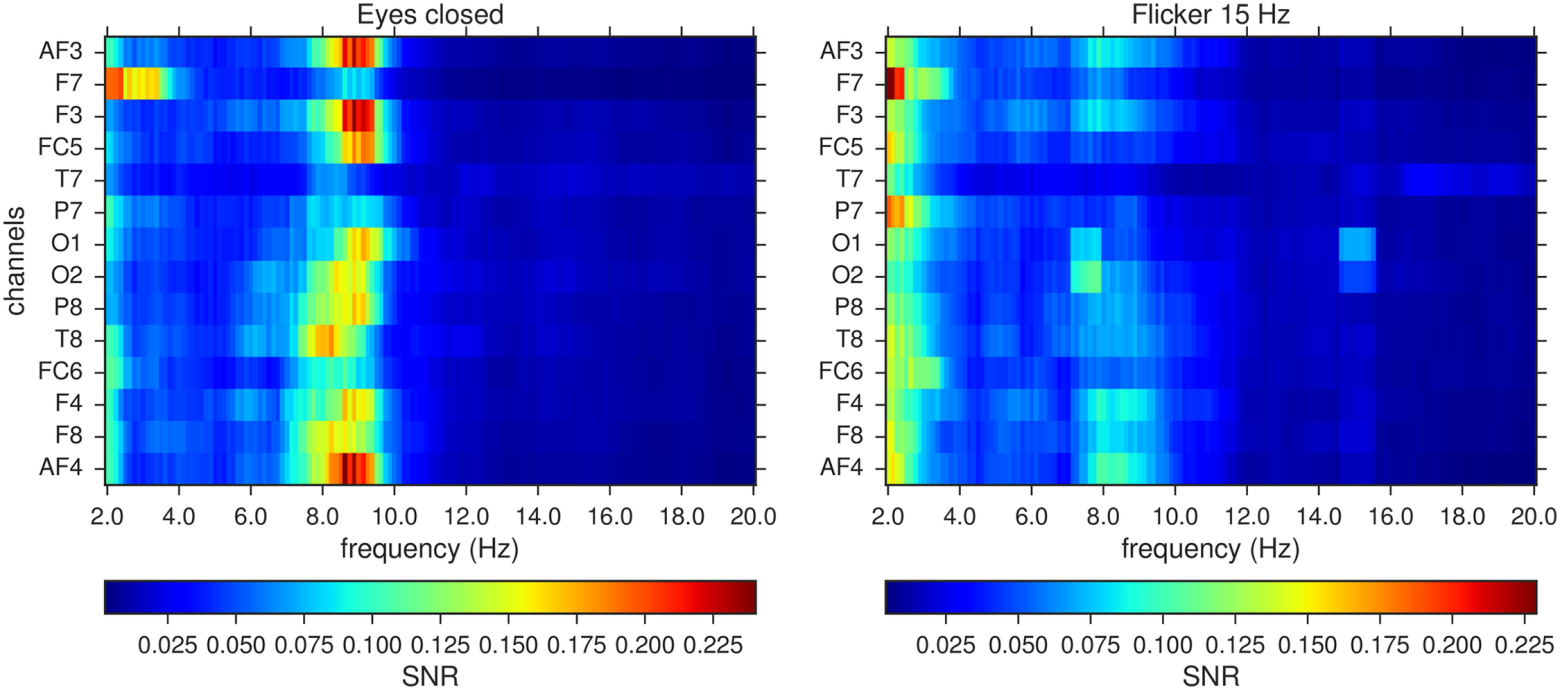
Mean SNR of the data corresponding to the two different experimental conditions for a subject in the study. (Left) Average over 6 realizations with eyes closed. (Right) Average over 6 realizations looking at a 15 Hz flickering screen.

As an example, Fig 3 shows the average SNR of each channel for both experiments obtained from one of the subjects. In the case of the eyes closed, one can observe a common delocalized, broadband high activity between 8 and 10 Hz approximately. In the case of the flicker, we can observe a common localized, narrow band rhythm oscillating at 15 Hz in several channels, in addition to the activity between 8 and 10 Hz. The activity at 15 Hz is greater in the occipital lobes. These lobes are fundamentally dedicated to the visual processing [30]. Other subjects show qualitatively similar spectra, specially in the case of the flicker. In the case of the eyes closed, we typically observe broadband high activity around the alpha frequency band, but the maximum activity is not exactly at the same frequency than for the case shown in Fig 3.

### Principal component analysis (PCA)

PCA is a standard method useful to reduce the dimensionality of Gaussian distributed data [28]. In general, a *n* × *m* matrix can be reduced to *k* × *m* with *k* = 1, 2, …, *n*. Our aim is to utilize this method to *compress* the time series corresponding to different sensor locations in a single one (*k* = 1), which is the collective rhythm. The goal of PCA is to express this global signal as the *eigensignal* with the greatest variance, which is obtained from a linear transformation of the raw data. This *eigensignal* is the one that retains more information from the original data. The alternative for non-Gaussian distributed data is independent component analysis (ICA) [31]. In our case, we checked that the results for PCA and ICA are equivalent since the first principal components are also independent.

### Phaser algorithm

The phaser algorithm applies to the estimation of a global phase from multidimensional data produced by a locked system of coupled oscillators [29]. The Phaser algorithm was originally applied to construct a global phase from the Hopf oscillator model and cockroach locomotion synthetic and empirical data in the body frame of reference [29]. This method turned out to be successful for the example of the cockroach locomotion but it is not clear if it is suitable in the case of EEG data. The main difference between the cockroach locomotion and EEG measurements is the measurement noise, which is higher in EEG data. In addition, the channels in the EEG data can have very different SNR and estimated number of cycles, making the phase inference more difficult. We note that the phaser estimation algorithm uses similar mathematical concepts to a previous work carried out by Kraleman *et al* [32], however the targets are completely different.

Although the algorithm can be also pre-trained and used with novel data, we only consider training data to fit a phase estimator. An implementation of the algorithm has been made available [33] by the original authors, and consists of the following steps (1-4):

1. (Metrization) Transform measurements to z-scores with equal variance [34]. These scores, *z*_*j*_, are defined such that ||*z*_*j*_|| is the Mahalanobis distance [35] of the (Gaussian) covariance matrix, *C*_*X*_, of measurements *x*_*j*_ − 〈*x*_*j*_〉:
  $$\begin{array}{c} z_{j}\equiv C_{X}^{-1/2}\left(x_{j}-\left\langle x_{j}\right\rangle \right)=U^{T}\Lambda^{-1/2}U\left(x_{j}-\left\langle x_{j}\right\rangle \right), \end{array}$$ (1)
  where *C*_*X*_ = *U*^*T*^Λ*U* and small bold letters denote time series represented as column vectors. The scores are linearly uncorrelated because its covariance matrix is the identity. Notice that diagonalization of *C*_*X*_ can be done through SVD of the centered original data, and normalization by $C_{X}^{-1/2}$ transforms the original data into time series with uncorrelated measurement noise and similar variance.
2. (Protophases) Compute the individual instantaneous phases for each score time series using the Hilbert transform [36].
3. (Series correction operator) Apply Fourier series based correction to the individual phases. This step is more robust to measurement noise in [29] than in [32]. This process is denoted by the series correction operator, P, which applies to a single phase *θ*_*i*_(*t*) and corrects systematic errors in *θ*_*i*_(*t*). The entire process of approximating the actual phase, *ϕ*_*i*_, from *θ*_*i*_ is written as $\hat{\phi_{i}}=P\left[\theta_{i}\right]$.
4. (Combine multiple estimates) Combine the individual phase estimates into a single global phase using PCA. Once the individual phases have been estimated using the series correction operator, they are combined into a single, improved global phase, $\hat{\phi }\approx \phi$, of the phase-locked system with actual phase *ϕ*. The combination has the purpose of improving the SNR. First, an analytic signal with constant amplitude envelope is reconstructed for each coordinate as ${\hat{q}}_{2j}\left(t\right)+i{\hat{q}}_{2j+1}\left(\theta \right)$, so that
  $$\begin{array}{ccc} {\hat{q}}_{2j}\left(t\right) & \equiv & \rho_{j}\sin\left({\hat{\phi }}_{j}\left(t\right)\right),\\ {\hat{q}}_{2j+1}\left(t\right) & \equiv & \rho_{j}\cos\left({\hat{\phi }}_{j}\left(t\right)\right), \end{array}$$ (2)
  where *ρ*_*j*_ has been previously obtained from the time averaged amplitude envelope of the corresponding time series, i.e.
  $$\begin{array}{c} \rho_{j}=\left\langle \right|z_{j}\left(t\right)+iH\left[z_{j}\left(t\right)\right]\left|\right\rangle , \end{array}$$ (3)
  where *z*_*j*_(*t*) are the z-scores of the original time series. The magnitudes *ρ*_*j*_ are expected to be higher when ${\hat{\phi }}_{j}$ are closer to the actual phase *ϕ*.
  Therefore, since ${\hat{q}}_{2j}\left(t\right)$ and ${\hat{q}}_{2j+1}\left(t\right)$ are orthogonal, we fill a data matrix $\hat{Q}$ with the time series ${\hat{q}}_{j}\left(t\right)$ organized in columns to perform PCA accounting for small phase shifts. The first two principal components, ${\hat{v}}_{\tilde{1}},{\hat{v}}_{\tilde{2}}$ of $\hat{Q}$ are used to obtain two orthogonal projections, which provide a phase estimation that is also series-corrected with the operator P, i.e.
  $$\begin{array}{c} \hat{\phi }=P\left[\arg\left(\hat{Q}\left({\hat{v}}_{\tilde{1}}+i{\hat{v}}_{\tilde{2}}\right)\right)\right]. \end{array}$$ (4)

### Kosambi-Hilbert torsion (KHT)

Schwabedal and Kantz [27] discussed the possible benefits of improved phase inference and proposed a method called Kosambi-Hilbert torsion (KHT), which optimally infers the phase dynamics of a collective rhythm. KHT has the same target than the Phaser and PCA algorithms for k = 1 applied to collective rhythms. KHT is a transformation based on methods proposed by Kosambi [37] and Hilbert, hence its name. It maximally amplifies the SNR of an oscillatory signal which is supposed to be common in all channels, trying to avoid spurious phase slips.

Schwabedal and Kantz have made available an implementation of the KHT [38], which consists of the following steps (1-6):

(Reference phase) Choose a reference channel, which will lock the phase. We assume that the phase obtained from the reference channel will be well defined and will be similar to the (unknown) real collective phase. In our case, we use the channel with the largest SNR as a reference for the KHT.
(Normalization) Compute the noise intensity $\sigma_{noise,j}^{2}$ for each channel and use it to normalize each channel *x*_*j*_ ↦ *x*_*j*_/*σ*_*noise*,*j*_. This normalization choice makes the SNR to be the optimization objective of this method.
(Extended phase space) Construct the data matrix *X* = (*x*_1_, *x*_2_, *H*(*x*_2_), …, *x*_*n*_, *H*(*x*_*n*_)), where each component is a column vector containing the time evolution. *H*(*x*_*j*_) denotes the Hilbert transform of the channel *x*_*j*_, and *n* is the number of channels. Notice that *H*(*x*_1_) is not present here.
(Filter) Bandpass filter *X* by columns to obtain *X*^*f*^ at the desired frequency and bandwidth. In our case, we used sharp bandpass filters.
(SVD) Compute *V* using singular value decomposition (SVD) [39] on the filtered data matrix $X^{f}=U\Sigma V^{\text{T}}$, where *U* is an *m* × *m* real or complex unitary matrix, Σ is an *m* × *n* rectangular diagonal matrix with non-negative real numbers on the diagonal, and *V* is an *n* × *n* real or complex unitary matrix. This problem is equivalent to the diagonalization of the covariance matrix $C\equiv {\left(X^{f}\right)}^{\text{T}}X^{f}=V\Sigma^{\text{T}}\Sigma V^{\text{T}}$. As a convention, the greatest eigenvalue is the first element of the diagonal matrix $\Sigma^{\text{T}}\Sigma$, and the corresponding first component of the rotation matrix *V*, as in PCA, is the direction that retains the greatest variance.
(Collective rhythm estimation) Apply the orthonormal rotation *V* to *X* to get an estimation of the collective signal: *y*(*t*) = (*VX*)_*t*1_, i.e. the original extended data matrix *X* is rotated in the direction that retains the greatest variance of the filtered matrix *X*^*f*^. We keep only the first column of the result.

In summary, the KHT algorithm computes the optimum torsion that projects a group of signals onto a component with the largest SNR. This optimum projection is computed at the extended phase space trajectory of the filtered signals and applied back to the original (unfiltered) signals.

### Definition of the signal-to-noise ratio

The signal-to-noise ratio (SNR) is a measure of the level of signal compared to the level of background noise of a time series. Given a time series, the corresponding SNR is computed as the signal variance divided by the noise variance,

$$\begin{array}{c} SNR=\frac{\sigma_{signal}^{2}}{\sigma_{noise}^{2}}. \end{array}$$ (5)

A high SNR indicates high precision data. The noise variance depends on the definition of noise, which in our case and for an arbitrary signal we define using bandpass filters at different frequencies and a given bandwidth. The level of noise then corresponds to the out-of-band variance, following the recommendations in [27]. The procedure to compute the SNR is the following:

1. Given a time series *x*(*t*), select the desired center frequency and bandwidth to apply a bandpass filter to *x*(*t*) and obtain *x*^*f*^(*t*), which is the filtered signal.
2. Compute the signal variance at the given center frequency, *f*_*c*_, as $\sigma_{signal}^{2}$ = var[*x*^*f*^(*t*)].
3. Compute the noise variance at the given center frequency as $\sigma_{noise}^{2}$ = var[*x*(*t*) − *x*^*f*^(*t*)].
4. Compute the SNR, see Eq (5).

Here, everything that is not the signal within a given frequency range is considered to be noise. This procedure can be repeated for several center frequencies in the desired range to obtain the spectrum *SNR*(*f*_*c*_).

### Extraction of an instantaneous phase

The analytic signal is defined as *y*_*a*_(*t*) = *y*(*t*) + *iH*(*y*(*t*)), where *H*(*y*(*t*)) is the Hilbert transform of *y*(*t*). This analytic signal can also be written as *y*_*a*_(*t*) = *A*(*t*)exp(*iϕ*(*t*)), where *A*(*t*) is the amplitude envelope and *ϕ*(*t*) = arg[*y*_*a*_(*t*)] is the instantaneous phase [36]. If *y*(*t*) is an estimation of a global signal, then *ϕ*(*t*) is an estimation of a global phase.

### Addressing non-stationarity

Variations of the signal and noise amplitudes, artifacts, or even brief disconnections are not features of periodic or quasi-periodic data. These potential drawbacks can make the mean and variance to be different at two different temporal windows, affecting in turn the performance of SVD applied to either KHT, phaser or PCA. Typically, EEG data is non-stationary and the SNR changes in time. To deal with this issue, we use a windowing technique, computing the global signal with the corresponding method using 20 oscillations per window and an overlap of 10 oscillations per window. Then, the resulting signals obtained for each window are smoothly concatenated, as in [27].

## Results

Our aim is to extract a global signal that represents the underlying dynamics of the system out of the whole set of measured channels. To that end, we have evaluated the performance of the three methods described above, namely the KHT, Phaser and PCA. The computation of the global signal allows us to evaluate the corresponding SNR curves, which may have different shapes for the different experiments and subjects. Other quantities relevant to this study are the global phase and the extracted number of cycles. In fact, a good criterion to evaluate which collective rhythm has the best phase estimation is to choose the best method in terms of the SNR. We have checked that the collective rhythm extracted from the time series is more accurate when choosing the method that generates the best approximation for the phase of the temporal signal. A better phase approximation for the measurement of the number of cycles implies that forward or backward spurious phase slips are reduced to a minimum. Therefore, the SNR spectrum corresponding to a better phase extraction method is more accurate. In the following we compute the global phase and the SNR curves, discussing the main results obtained from the experimental data. For the sake of clarity in the presentation, we show the results for a single subject in the first two sections and for all subjects in the third Results section.

### Evaluation of the global phase

The global phase is the instantaneous phase of the global signal that we estimate from the collected experimental data. As described in the Methods section, this phase is obtained from the analytic signal of the estimated common rhythm. We show results for a single subject in this section. Other subjects present similar results in terms of the properties of the phase extraction methods.

**Fig 4:**
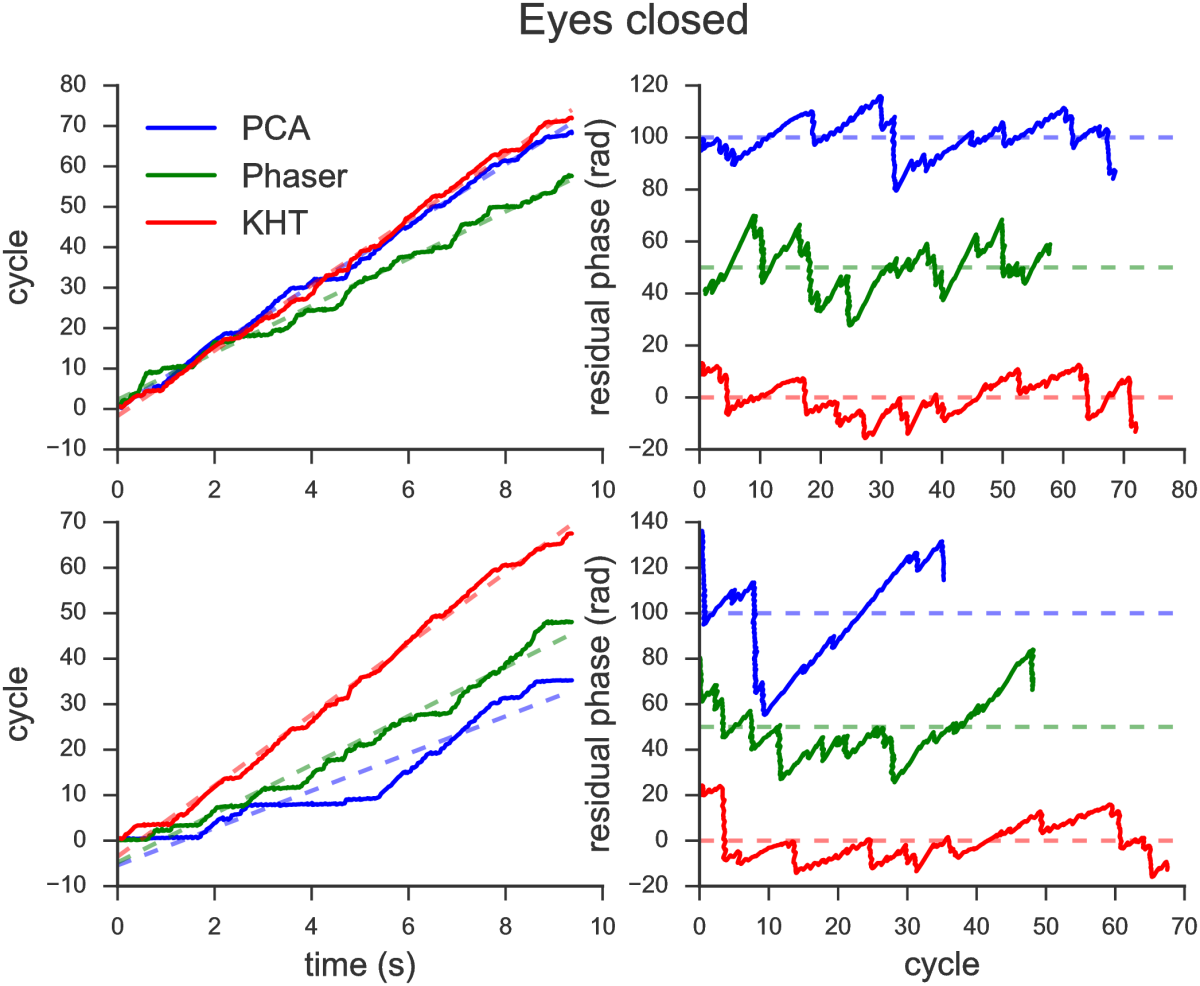
Evaluation of the global phase for the eyes closed experiment. (Left) Instantaneous phases and linear fits. (Right) Residual phases as functions of the corresponding cycles. (Top) Only 5 channels have been used in the analysis. (Bottom) All available channels have been used.

**Fig 5:**
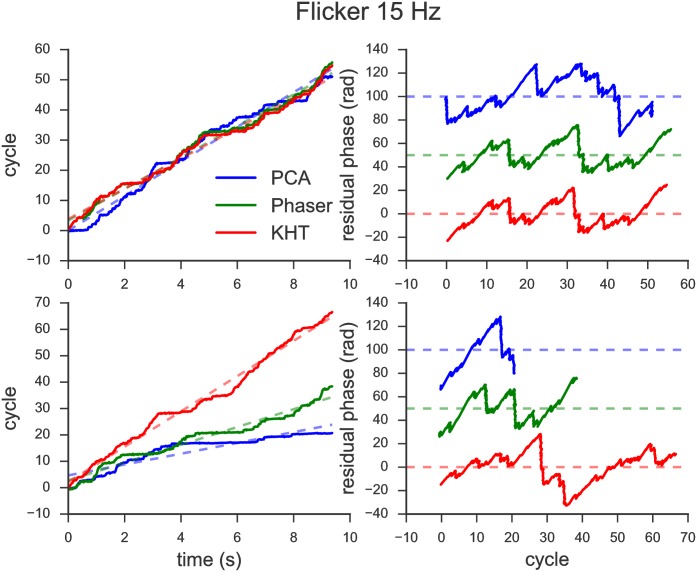
Evaluation of the global phase for the 15 Hz flickering experiment. (Left) Instantaneous phases and linear fits. (Right) Residual phases as functions of the corresponding cycles. (Top) Only 2 channels have been used in the analysis. (Bottom) All available channels have been used.

Figs 4 and 5 show the evaluation of the global phase for the two experimental conditions. On the one hand, in the left panels of these two figures, we represent the extracted phases (solid lines) and the corresponding linear regression (dashed lines) as a function of time for the estimations obtained from the three methods: PCA (blue), Phaser (green) and KHT (red). The phase estimation has been obtained from 10 seconds data sets in both cases: eyes closed (Fig 4) and flicker at 15 Hz (Fig 5). Also, notice that top and bottom panels are different. In the bottom panels, the phase is approximated using all available channels, while in the top panels, it is approximated using 5 (2) channels in the case of the eyes closed (flicker). We have manually picked the number of channels in each scenario such that the attributes of the methods are better represented.

On the other hand, in the right panels of Figs 4 and 5, we plot the residual phases as a function of the cycles for each of the three methods, which have been shifted from each other for clarity. The residual phases are computed as the difference between each phase and the corresponding linear fit, i.e. it evaluates deviations from an ideal model with constant angular frequency. The residual phase allows us to compare the total number of cycles and similarities between different residual phases.

Comparing Figs 4 and 5, we note that the KHT is the most consistent method when the number of data channels used in the analysis is modified. Recall that the results shown in the top and bottom panels are computed for a different number of channels. Thus, the KHT is the most consistent method since the slope of the extracted phase is the most similar between top left and bottom left panels for both experiments. Moreover, the residual phases are also the most similar when comparing top right and bottom right panels for the KHT estimation. These findings illustrate that this method is more robust when adding data channels with a lower SNR.

In contrast, comparing PCA and Phaser does not seem so straightforward as in the case of the KHT just by looking at Figs 4 and 5. We observe that the signal recovered from the phase obtained using the Phaser algorithm does not provide good results compared to PCA and KHT in most cases. The only case in which we obtain comparable signals from the three methods is for the phases shown in the top panel of Fig 5. This is because the main rhythm present in these two channels have high enough SNR and the detected number of cycles is very similar for each channel. Nevertheless, when data is not selected manually, it will be by chance that these conditions hold.

After careful evaluation, we have discarded the Phaser algorithm to analyze EEG time series. Therefore, the results using this method are omitted in the next sections. We observed that in general a simple PCA works better than the Phaser algorithm for our data. The latter only works correctly when the number of cycles of the different channels is very similar, as in Figs 4 and 5 top left panels, for which all manually selected data channels have almost the same number of oscillation cycles.

### Evaluation of the signal-to-noise ratio

Given a time series, the corresponding SNR is computed as the signal variance divided by the noise variance (see Eq (5)). For the estimated (KHT and PCA) collective rhythms, one can also compute the SNR enhancement Δ*SNR* = *SNR*_*global*_/∑_*j*_ *SNR*_*j*_, where *SNR*_*global*_ is the *SNR* of the estimated global signal and *SNR*_*j*_ are the corresponding SNR of the individual channels Thus, Δ*SNR* is a normalization of *SNR*_*global*_ weighted by the contributions from all the channels.

As mentioned earlier, we expect a higher activity in the alpha band for the eyes closed experiment. The alpha activity (8-12 Hz) is higher when the subject is awake and relaxed with eyes closed, but such activity is attenuated when the subject is with eyes open, making mental efforts or asleep [40]. For the flicker at 15 Hz, we expect an additional and localized activity at 15 Hz [41].

Here, we use the SNR as a metric to compare the phase estimations from the PCA and KHT methods. Since the real phase is here unknown, we rely on the SNR to estimate the quality of the different methods. Given that the bandwidth of the electrodes is 43 Hz and the sampling rate of the Emotiv EPOC is 128 samples per second, we restrict ourselves to the computation of the phase for frequencies below 20 Hz.

**Fig 6:**
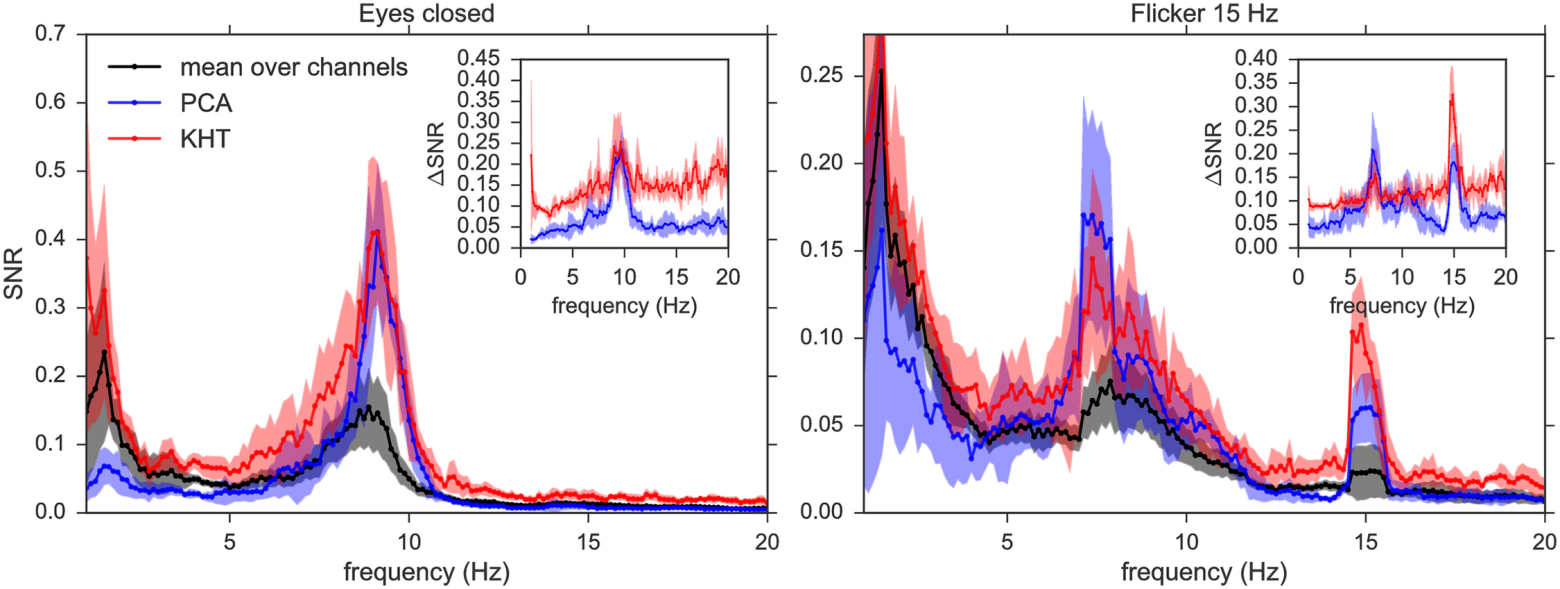
Single subject mean SNR and SNR enhancement, and their standard deviations of the PCA (blue) and KHT (red) collective rhythms. The mean SNR over channels and experiments and its standard deviation is also shown in black/grey. PCA results correspond to the highest SNR *eigensignal*, which in this case is the second principal component. The results are an average over the six realizations for both experiments: eyes closed and flicker at 15 Hz, and we analyze 10 seconds for each realization. The parameters are: 14 channels, 128 samples per second, 1 Hz bandwidth for each center frequency (KHT) and 20 oscillations per window.

In Fig 6 we show the SNR and its enhancement for eyes closed and flickering experiments for one of the subjects to illustrate the effect of PCA and KHT in the SNR spectrum. The SNR enhancement (Δ*SNR*), which is bounded between 0 (*SNR* = 0) and 1 (theoretical limit), is shown in the insets of Fig 6 for both eyes closed and flickering experiments. One can observe a slightly larger overall SNR enhancement using KHT (red) in contrast to PCA (blue). The Δ*SNR* reveals maximum enhancements at the peaks. In the case of the eyes closed, the peak around 9 Hz is quite similar for both the KHT and the PCA. In the case of the flicker, the peak at 15 Hz is enhanced by up to 16% using PCA and up to 33% using the KHT with respect to the theoretical maximum limit at the peak. Other subjects show qualitatively similar shapes of the SNR frequency curves. In general, the KHT gives a larger SNR enhancement at the frequencies of interest for most subjects.

Interestingly, the projection to obtain the optimum collective rhythm in the case of the PCA does not always correspond to the component with the largest variance. We find that the highest variance projection does not necessarily correspond to the *eigensignal* with highest SNR. This is a known issue of the PCA when it is applied to EEG signals [42]. Some studies in EEG-based BCI suggest methods to choose the appropriate principal components, e.g. linear discriminant analysis for a classification task [42] or higher-order statistics for the detection of steady-state visual evoked potentials [43]. In these cases the principal component with the largest variance is not the most relevant for the specific purpose. The reason is that for EEG signals with a low SNR, the variance of the signal of interest can be lower than that of the noise due to internal and external artifacts. Therefore, selecting the relevant PCA component is not straightforward in the case of EEG, and specially in the case of consumer grade headsets. Here, we use the second largest variance projection to plot the blue lines in Fig 6. This second component of the PCA turns out to correspond to the *eigensignal* with a highest SNR. Actually, using the highest variance projection in PCA we obtained a SNR curve similar to that of the mean over channels.

In Fig 6, we note that the KHT also extracts better other less relevant frequency bands which are not enhanced or are even lost using PCA. An example of such an enhancement is the activity around 5 Hz for the eyes closed experiment. The SNR computed from the PCA estimation drops below the mean SNR computed from the raw data (black), i.e. the activity is under-represented in this frequency band. In contrast, the SNR of the KHT estimation is enhanced.

**Fig 7:**
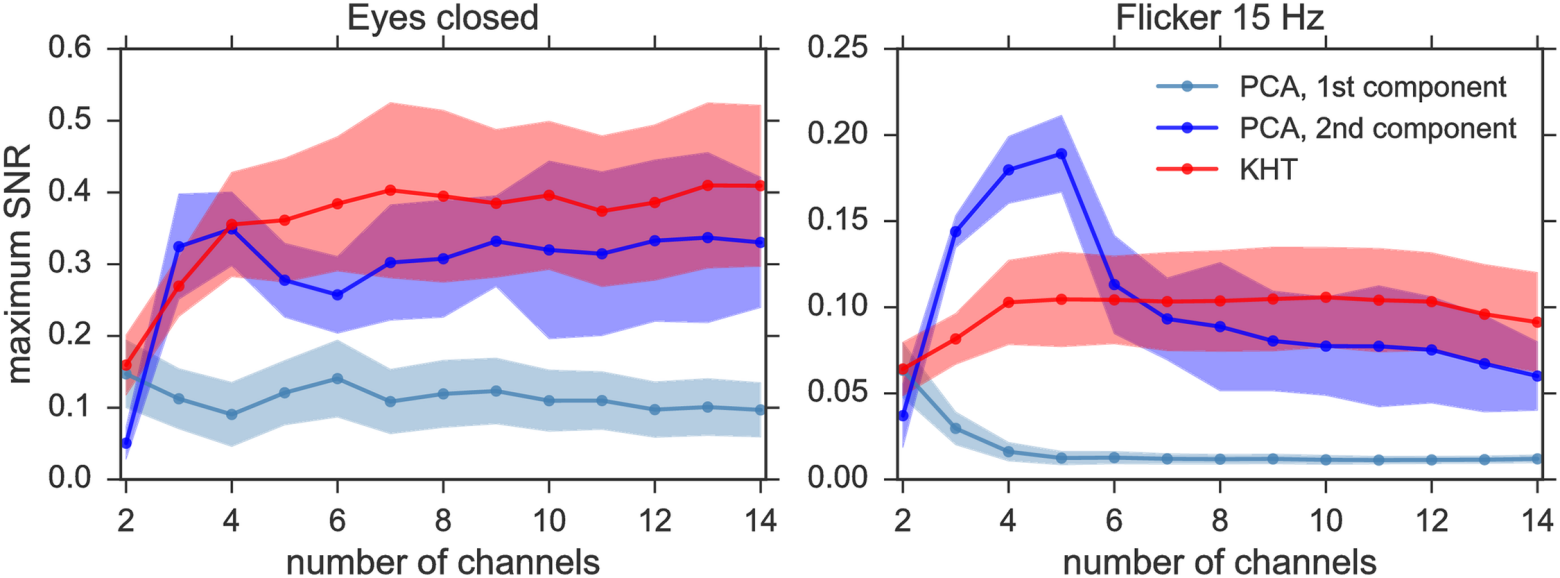
SNR for the eyes closed and flickering experiments. The parameters are the same as in Fig 6, changing the number of channels and evaluating the SNR of the estimated collective rhythms only at the peaks of interest. These collective rhythms have been estimated using PCA (using first and second largest variance projections) and KHT methods. For this subject, the peaks are located at 9 Hz in the case of eyes closed and 15 Hz for the flickering.

Fig 6 shows the results for all available (14) channels. Since the precise results depend on the number of data channels used in the analysis, we show in Fig 7 the SNR at the peaks of interest for different number of channels, added in decreasing order of SNR. For this subject, the peaks of interest are at 9 Hz in the case of eyes closed and 15 Hz for the flickering. As shown in Fig 7, the KHT provides in general a better phase estimation than PCA, while the order of magnitude of the obtained SNR is the same for both methods. Since the SNR is not the same for all channels, adding very noisy channels may sometimes decrease the SNR of the extracted collective rhythm. In this regard, it seems that the KHT is more robust to the addition of channels with lower SNR. It can be seen in Fig 7 that the SNR of the phase extracted using PCA indeed can present large variations when using a different number of channels.

**Fig 8:**
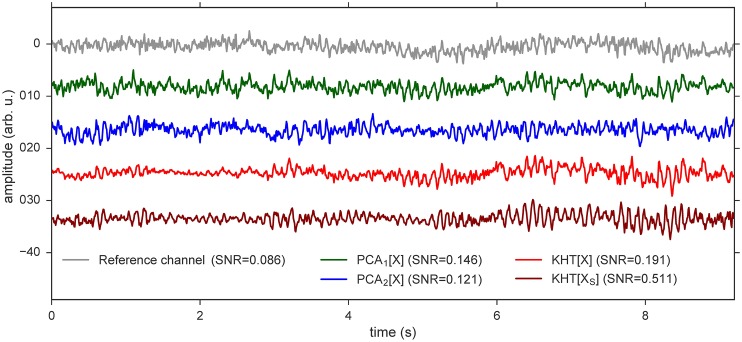
Several examples of estimations of collective rhythms from a data set with the eyes closed, which are shifted from each other for clarity. For each signal, the corresponding SNR for the frequency band 8.5–9.5 Hz is indicated in the legend. The PCA signals are computed by parts of 20 oscillations at 9 Hz and KHT signals are also computed at this frequency band (8.5–9.5 Hz). (Grey) Reference channel for the KHT estimations, this channel is the one with highest SNR. (Dark green) PCA estimation using the first principal component computed from the raw data. (Blue) PCA estimation using the second principal component computed from the raw data. (Red) KHT estimation. (Dark red) Example of a KHT estimation computed from time shifted data. We used 14 channels for the computation of all the collective rhythms, except for the top time series, which is the raw data of the reference channel.

Finally, we illustrate in Fig 8 the different global rhythms extracted using the procedures described above. In this example we use a 10 seconds eyes closed data set for the calculations and only 7 seconds are shown. The top time series is the raw signal of the reference data channel (grey) chosen for the computation of the KHT method (channel with highest SNR). From top to bottom, the second and third time series are the PCA estimations using the projection onto the first principal component (green) and the second principal component computed (blue). The fourth time series is the KHT computed from raw data (red) centered at 9 Hz with 1 Hz of bandwidth. In Fig 8, we note that for this example the best PCA estimation of the collective rhythm already has a good SNR (SNR = 0.146) but the original KHT yields a slightly better estimation (SNR = 0.191). The bottom time series in Fig 8 is the KHT computed from time shifted raw data (dark red), following a procedure that will be described in the next section.

### Enhancing the signal-to-noise ratio

In the previous section, we have seen an improvement in the SNR of the estimated collective rhythm using KHT compared to PCA. However, the ratios of improvement remain low. This is probably due to the fact that the experimental data does not contain major phase lags between the channels. Therefore, we explore here what happens when phase lags among time series are artificially added, and are not only due to inherent mismatches. In advance, we can already anticipate that the SNR of the collective signal extracted with PCA will typically degrade in presence of significant phase lags. But, how does higher phase lags affect the performance of KHT? To answer this question we shift in time all data channels using random uniform shifts and subsequently analyze the SNR. In this section, we show the results for the five subjects of the current study.

**Fig 9:**
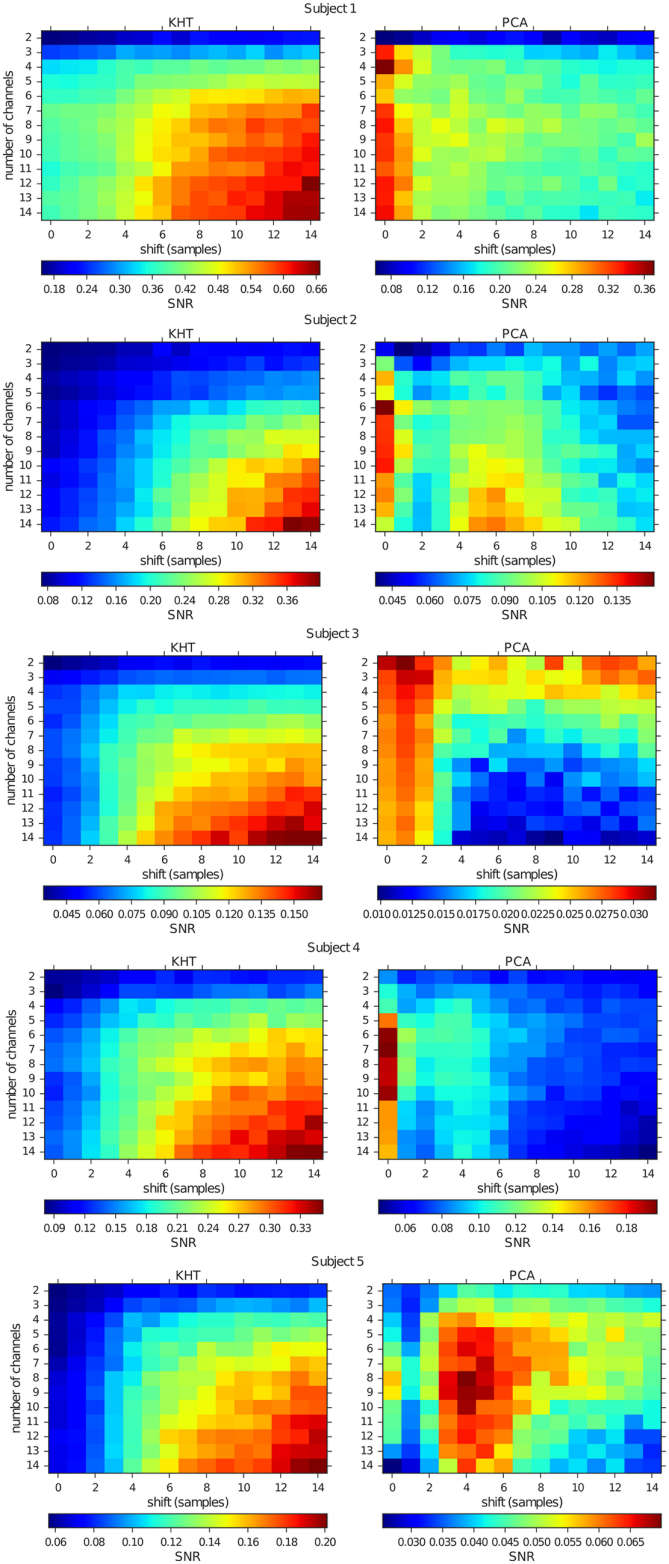
Mean SNR for the eyes closed experiments at the corresponding peaks in the alpha band computed from KHT (left) and PCA (right) collective rhythm estimations. The horizontal axis indicates the maximum time shift. This time shift is random uniform among time series and we obtain the SNR averaged over 30 random realizations and the corresponding experimental realizations. The vertical axis indicates the number of channels added in decreasing order of SNR used for the computation of both quantities.

**Fig 10:**
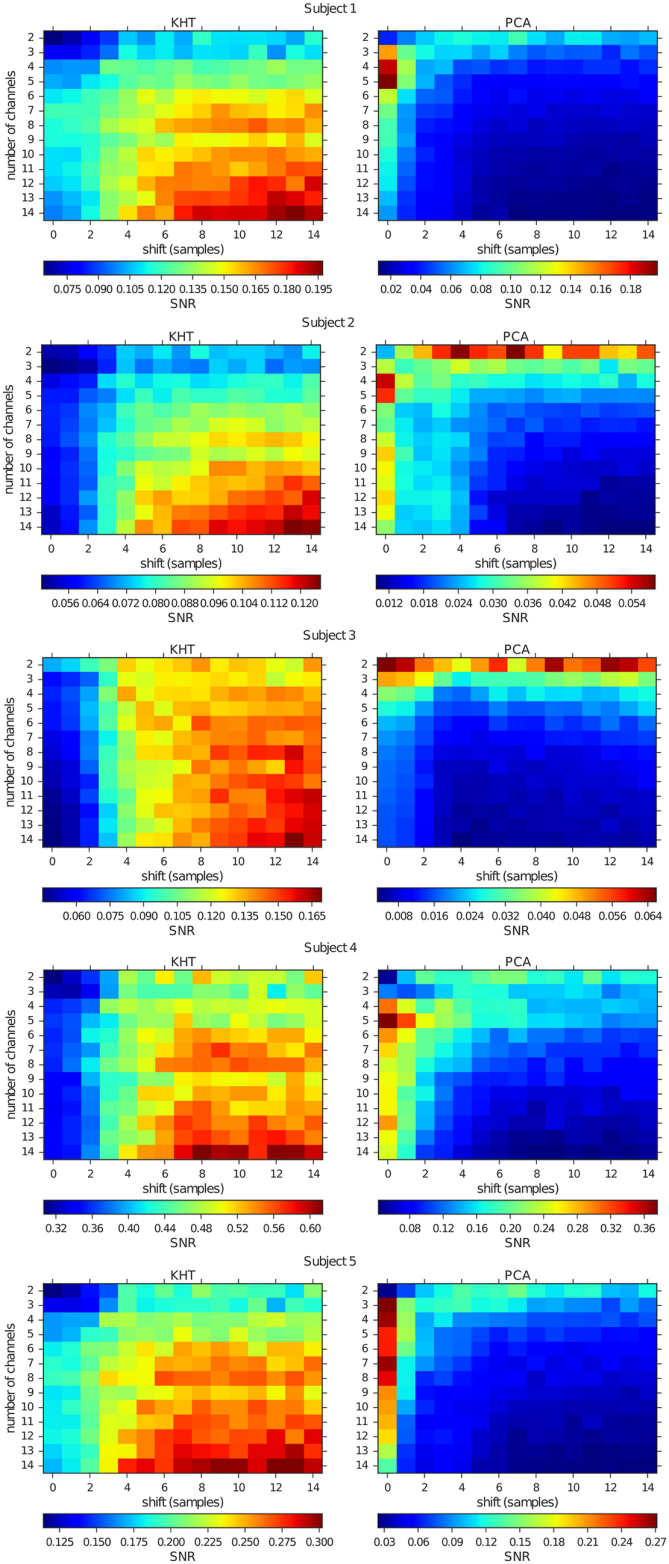
Mean SNR for the flickering experiments at the 15 Hz peak computed from KHT (left) and PCA (right) collective rhythm estimations. The horizontal axis indicates the maximum time shift. This time shift is random uniform among time series and we obtain the SNR averaged over 30 random realizations and the corresponding experimental realizations. The vertical axis indicates the number of channels added in decreasing order of SNR used for the computation of both quantities.

Figs 9 and 10 show the SNR at the peaks of interest for both experiments, varying the number of channels, added in decreasing order of SNR, and for different number of shifted samples. In the case of the eyes closed experiment, the analyzed peak is located near 9 Hz, but varies across different subjects, while in the case of the flickering we always analyze the 15 Hz peak. In these figures, we change the maximum number of shifted samples for each realization according to the number indicated in the horizontal axis. Each channel is shifted by a random number of samples within the allowed range [−max(*shift*_*j*_), max(*shift*_*j*_)] for *j* = 1, 2, …, 14. The horizontal axis in Figs 9 and 10 indicates the maximum shift allowed in each case.

One can observe in Figs 9 and 10 that for both experiments the results are very similar. In the case of PCA, the best SNR is obtained for low to intermediate number of channels and temporal shift. In contrast, when using the KHT, the best SNR is obtained for high number of channels and temporal shift. We note that the KHT estimation typically saturates at a maximum SNR as we increase the maximum temporal shift. In Table 1, we show the optimum number of channels and temporal shift associated to the best SNR for all the subjects in this study. It is apparent that the absolute SNR values vary across subjects but there is a clear trend towards the use of a high number of channels and a high temporal shift.

**Table 1:** KHT optimum cases extracted from the data represented in Figs 9 and 10 for the five subjects.

KHT optimum cases						
id	Eyes closed			Flicker 15 Hz		
	channels	shift (samples)	SNR	channels	shift (samples)	SNR
1	12	14	0.67	14	13	0.2
2	14	13	0.40	14	13	0.13
3	14	13	0.16	14	13	0.17
4	14	14	0.35	14	8	0.61
5	14	14	0.20	14	13	0.30

One could expect a priori that the SNR of the KHT remains approximately constant when the time shift between the data channels is changed, however the SNR actually increases. Our interpretation of these results rely on the fact that the KHT method aims at correcting phase lags between a reference channel and the rest of the channels. In this manner, the rest of the channels are phase-shifted in order to obtain in-phase oscillations. This shift is typically restricted to be smaller than half a period of the main oscillating signal. The correcting shift applied by the KHT aims at keeping the phases aligned. This procedure does not necessarily align the amplitudes when going back to the original signal space. For increasing time shifts, we have checked that the variance of the out-of-band signal decreases since the amplitudes lose correlation. At the same time the variance of the in-band signal increases, leading to the observed increase in the SNR.

In the bottom signal of Fig 8 a single realization for a single subject is shown, illustrating that the KHT computed from time shifted data yields an even better estimation (SNR = 0.511) of the global signal for this example. The SNR of the better estimation is 5.9 times higher than the SNR of the raw signal of the reference channel and 3.5 times higher than the SNR of the best PCA estimation.

## Discussion and outlook

Here, we compare the standard PCA to more recent approaches to extract a collective rhythm from phase-synchronized data. We observe that the KHT method improves the SNR of a collective EEG signal over the standard PCA. More specifically, we find this clear improvement when we add random phase lags (temporal shifts) among time series before using the KHT.

For the experimental data recorded with the eyes closed condition and using the KHT method, the quality of the extracted collective rhythm keeps improving as more channels are added to the analysis, even if the added channels have a lower SNR. In contrast, we find that using the PCA the best result is typically obtained by selecting only a few channels with the highest SNR.

For the experimental data recorded watching a flickering screen, the quality of the extracted collective rhythm using the KHT improves when channels with a lower SNR are added to the analysis. In contrast, we found that using only a few channels is the best choice when using the PCA. In the latter case, adding more channels with lower SNR typically makes the quality of the collective rhythm to start decreasing significantly.

Comparing all subjects and the two experimental conditions, we find a larger SNR for the KHT than for the PCA. The overall SNR enhancement when using all channels is larger in the case of the eyes closed experiment than in the flickering screen one. This is due to the fact that the signal is more distributed along the channels in the former case.

Here, we recorded EEG data for two experimental conditions in order to characterize the signal quality of a commercial “low-cost” headset (Emotiv EPOC). We show that the KHT method provides an improvement in the quality of the extracted collective rhythm. We argue that similar qualitative results are to be expected, in terms of the SNR improvement of a collective signal, using other EEG devices and in the presence of phase lags. This is a major advantage of the KHT over the PCA by the very own definition of the methods, independent of the EEG recording device. In this context, we also show that the introduction of an additional time-shift (or phase lag) to the original time series can enhance the extracted signal quality when using the KHT method. This finding applies to signals whose main frequency content is sustained over time.

As future work, we intend to test the performance of the KHT outcome for BCI tasks (e.g. visual stimuli or motor control) [44, 45]. The computational complexity of this method does not pose a problem in terms of computing power or computing time since it relies on the singular value decomposition. In this case, however, spatial filtering techniques are already extensively used [46, 47] and one would need to validate the KHT in front of such methods. Finally, we note that knowing the coefficients of the optimum torsion, the phase lags between the different channels can be easily recovered. Thus, the KHT can be used to obtain reliable estimations of the real phase lags between brain areas, also if a professional EEG device is used.
